# Light intensity and spectrum affect metabolism of glutathione and amino acids at transcriptional level

**DOI:** 10.1371/journal.pone.0227271

**Published:** 2019-12-31

**Authors:** Dávid Toldi, Mónika Gyugos, Éva Darkó, Gabriella Szalai, Zsolt Gulyás, Krisztián Gierczik, András Székely, Ákos Boldizsár, Gábor Galiba, Maria Müller, Livia Simon-Sarkadi, Gábor Kocsy

**Affiliations:** 1 Department of Food Chemistry and Nutrition, Szent István University, Budapest, Hungary; 2 Doctoral School for Food Sciences, Szent István University, Budapest, Hungary; 3 Agricultural Institute, Centre for Agricultural Research, Martonvásár, Hungary; 4 Festetics Doctoral School, Georgikon Faculty, University of Pannonia, Keszthely, Hungary; 5 Institute of Biology, Department of Plant Sciences, University of Graz, Graz, Austria; University of Hyderabad School of Life Sciences, INDIA

## Abstract

The effects of various light intensities and spectral compositions on glutathione and amino acid metabolism were compared in wheat. Increase of light intensity (low—normal—high) was accompanied by a simultaneous increase in the shoot fresh weight, photosynthetic activity and glutathione content. These parameters were also affected by the modification of the ratios of blue, red and far-red components (referred to as blue, pink and far-red lights) compared to normal white light. The photosynthetic activity and the glutathione content decreased to 50% and the percentage of glutathione disulfide (characterising the redox state of the tissues) in the total glutathione pool doubled in far-red light. The alterations in the level and redox state of the antioxidant glutathione resulted from the effect of light on its synthesis as it could be concluded from the changes in the transcription of the related genes. Modification of the light conditions also greatly affected both the amount and the ratio of free amino acids. The total free amino acid content was greatly induced by the increase of light intensity and was greatly reduced in pink light compared to the normal intensity white light. The concentrations of most amino acids were similarly affected by the light conditions as described for the total free amino acid content but Pro, Met, Thr, ornithine and cystathionine showed unique response to light. As observed for the amino acid levels, the expression of several genes involved in their metabolism also enhanced due to increased light intensity. Interestingly, the modification of the spectrum greatly inhibited the expression of most of these genes. Correlation analysis of the investigated parameters indicates that changes in the light conditions may affect growth through the adjustment of photosynthesis and the glutathione-dependent redox state of the tissues. This process modifies the metabolism of glutathione and amino acids at transcriptional level.

## Introduction

Alterations in the light conditions influence many physiological processes in plants [1–3]. Intensity and spectral composition of light are characterised by spatial (latitude, altitude) and temporal (daily, seasonal) changes. The reduced light availability with increasing latitude is associated with a proportional increase in the amount of diffuse blue light and a decrease in the red/far-red (R/FR) ratio. The ratio of blue light will also be greater at higher altitudes and the R/FR ratio decreases during sunset.

Light environment determines the growth and development of plants because of its effect on metabolism. The rapid stem elongation is a well-known symptom of light deficiency. Regarding the light quality, negative effect of blue light on plant growth was shown in Scots pine (*Pinus sylvestris* L.) since elongation of the seedlings was increased by the removal of blue light [4]. Inhibitory effect of blue light on plant growth is supposed to be a general phenomenon as recently it has been reported both in the cases of barley and wheat, respectively [5,6]. Illuminating *Chrysanthemum* plants with low R/FR ratio (0.4 or 0.7 instead of 2.4) LED lights at dusk had similar effect without influencing dry weight [7]. The effect of light on growth and other physiological processes of plants is mediated by the red/far-red-absorbing phytochromes and blue light-sensing cryptochromes, both directly contacting the phytochrome-interacting factors, which regulate many downstream genes [8–10]. In the activation of cryptochromes, the light-dependent redox changes also participate [11].

The primary biochemical effect of light quality and quantity is reflected in the changes of photosynthesis [1,12]. If the light intensity is higher than optimal or when under stress, the electron acceptor oxidised nicotinamide adenine dinucleotide phosphate (NADP^+^) will not be available in sufficient amount for photosynthesis because of the reduced rate of Calvin cycle. The higher ratio and intensity of blue or red lights will also increase the electron flow through PSI and PSII since the absorption maximum of chlorophylls is in this spectral range. Low R/FR ratio decreased photosynthesis, the amount of photosynthetic pigments, water use efficiency and biomass as well but increased stomatal conductance, transpiration, ethylene levels and stem height in *Oenothera biennis* [13].

Light-induced changes in electron transport rate may lead to a greater formation of reactive oxygen species (ROS), the level of which is controlled by the antioxidants [14–16]. The removal of excessive H_2_O_2_ was ensured by the accumulation of ascorbate (AsA) and glutathione (GSH) in chloroplasts, peroxisomes and cytosol in *Arabidopsis* leaves where the activity of catalase also increased in high light intensity [17]. The increase in light intensity with growing altitude was accompanied with greater total antioxidant content mainly due to the higher AsA concentration in alpine plants [18]. In addition, the levels of the antioxidants exhibited diurnal rhythms with maximum values during midday and minimum ones during the night, which further corroborates the relationship between light intensity and antioxidant levels [18]. The increase in the ratio of blue light with the increasing altitude can also affect antioxidants. This assumption was confirmed under controlled condition in leaves and roots of *Rehmannia glutinosa* since additional blue light (besides the basic illumination with white light) increased total antioxidant capacity and the activity of several antioxidant enzymes (superoxide dismutase, catalase, ascorbate peroxidase, glutathione peroxidase) [19]. Growth under low R/FR ratio (0.2 instead of the normal 1.1) corresponding to the light conditions of ‘shade’ leaves resulted in decreased AsA and GSH contents, activity of antioxidant enzymes and respiration rates in the leaves of *Phaseolus vulgaris* [20].

Light–induced redox changes also influenced the amino acid metabolism [3,21]. The amino acid levels were greater in light conditions than in dark conditions in poplar (*Populus tremula* × *Populus alba*) and the increased GSH level was associated with greater concentration of several amino acids in transgenic plants [22]. The amount of free amino acids was increased by high light intensity and decreased by low light intensity in *Chlamydomonas* [23]. The reduction of R/FR ratio (from 1.1 to 0.08) did not affect the Ala, Asp, Asn, Glu and Gly concentrations in the internode of sunflower [24]. The positive effect of blue light on Pro content was observed in *Solanum lycopersicum* L., but the involvement of the redox changes in this process was not demonstrated [25]. Blue light induced a greater increase in the amount of free amino acids than red light during postharvest treatment in tomato fruits as well [26]. The promoting effect of blue light on amino acid accumulation was also demonstrated in young barley plants [5]. Although these studies indicate the effect of light conditions on free amino acid levels, they did not give a widespread comparison of the influence of various light intensities and spectral conditions on them and did not investigate the transcription of the related genes.

In our earlier studies, the effect of fluorescent and LED lighting on metabolism and yield was compared in the flag leaves and seeds of wheat [6]. The aim of the present experiments was the comparison of growth, photosynthetic activity, glutathione and amino acid metabolism, as well as the expression of the related genes under various light intensities and spectral compositions (after changes in the ratios of blue, red and far-red lights) at seedling stage in order to see how these parameters are adjusted to the changing light conditions in wheat. These experiments can also answer the question whether light intensity and spectrum affect metabolism of glutathione and amino acids at transcriptional level.

## Materials and methods

### Growth conditions and treatments

Seeds of the bread wheat (*Triticum aestivum* L. ssp. *aestivum*) variety Chinese Spring were germinated for 1 day at 25 °C, 3 days at 4 °C and 2 days at 25 °C between wet filter papers. The seedlings were grown on half-strength Hoagland solution [27] for 10 days at 20/17 °C (day/night temperature) and 75% relative humidity with 16 hours illumination. Three various light intensities and three various spectral compositions were applied in two identical plant growth chambers (Conviron PGR15; Controlled Env., Ltd., Winnipeg, Canada) divided into three parts (shown in S1 Fig). The light modules were equipped with a continuous wide spectrum LED (Philips Lumileds, LXZ2-5790-y) and three narrow spectrum LED armatures with the dominant wavelengths of 448 nm (Philips Lumileds, LXZ1-PR01), 655 nm (Philips Lumileds, LXZ1-PA01) and 750 nm (Edison Edixeon, 2ER101FX00000001). Different intensities of white light (50, 250, 500 μmol/m^2^/s) were used and called as low, normal and high light (S1 Fig). Studying the effect of light quality, three spectral conditions with different blue/red and R/FR ratios were used, which are referred to as blue, pink and far-red lights (S1 Fig). In the latter experiments, the photosynthetically active radiation was 250 μmol/m^2^/s and the spectrum of the basic white light was modified by the various combinations of the above-mentioned light sources. Three independent experiments were conducted. The determination of growth (n = 12 in each experiment) and photosynthetic parameters (n = 10 in each experiment) and sampling for the biochemical and molecular biological measurements (n = 3 in each experiment, each sample contained the mixture of the leaves taken from 4 plants) were performed on the same day, in the middle of the photoperiod in order to exclude the effects of daily rhythms.

#### Analysis of photosynthetic pigments and photosynthetic activity

After the extraction of 200 mg leaf segments in 80% acetone, chlorophyll *a* and *b*, and the carotenoid contents were also measured by a Cary-100 UV-Vis spectrophotometer (Varian, Middelburg, Netherlands) using the method of Lichtenthaler [28].

For the characterisation of the photosynthetic activity of PSII, the effective quantum yield, Y(II), and the electron transport rate [ETR, [29]] were determined from ten intact attached leaves in each light conditions using a PAM-2000 fluorometer (Walz, Effeltrich, Germany).

#### Determination of thiol and amino acid contents

The thiols were determined in 200 mg leaf sample as described by [30], according to the method of [31]. They were separated by reverse-phase HPLC (Waters, Milford, MA, USA) and detected by a W2475 scanning fluorescence detector (Waters, Milford, MA, USA). The redox state of cysteine and glutathione were characterised by the percentage of their oxidised forms (cystine: CySS, oxidised glutathione: GSSG) in the total thiol pools [CySS% = CySS/(Cys+CySS)x100; GSSG% = (GSSG/(GSH+GSSG)x100]

For the analysis of free amino acids, leaf samples of 300 mg fresh weight were extracted with 2 ml cold 10% trichloroacetic acid as described by Gulyás et al. [32]. The free amino acids were detected by an automatic amino acid analyser (Ingos Ltd., Czech Republic) equipped with an Ionex Ostion LCP5020 cation-exchange column (22 × 0.37 cm). They were separated by stepwise gradient elution using a Li^+^-citric buffer system (Ingos Ltd., Czech Republic). Colorimetric detection was achieved at 570 nm and 440 nm (for Pro) after post-column derivatisation with ninhydrin reagent.

#### Investigation of the mRNAs by qRT-PCR

Total RNA was extracted from the leaves with the Direct-zol^™^ RNA Miniprep Kit (Zymo Research) as described by the manufacturer. Reverse transcription was carried out using M-MLV Reverse Transcriptase and oligo(dT)_15_ primer (Promega) according to the instructions of the manufacturer. The gene expression levels were measured by real-time qRT-PCR using a CFX96 thermocycler (Bio-Rad) with previously published and custom-made primers (S1 Table). Studied genes related to glutathione metabolism: adenosine phosphosulfate reductase (*APSR*, synthesis of the glutathione precursor, Cys), ascorbate peroxidase1 (*APX1*, reduction of oxidised glutathione in the ascorbate-glutathione cycle)), glutathione reductase (*GR*), glutathione synthase2 (*GS2*), glutathione *S*-transferase (*GST*, conjugation of glutathione with peroxides and xenobiotics) and *O*-acetylserine (thiol) lyase (*ASTL*, Cys synthesis). Studied genes related to amino acid metabolism: arginine decarboxylase (*ADC*), argininosuccinate lyase (*ArgSUL*), aspartate transaminase (*AspTA*), glutamate dehydrogenase (*GluDH*), nitrate reductase (*NR*), ornithine aminotransferase (*OrnATF*), pyrroline-5-carboxylate reductase (*P5CR*, Pro synthesis) and serine hydroxymethyltransferase (*SerHMTF*). The values of the relative gene expression changes were calculated as described earlier applying *Ta30797* gene reference, which is similar to phosphogluconate dehydrogenase protein [33].

#### Statistical analysis

Biochemical data from three independent experiments were evaluated and standard deviations are indicated in the figures. Statistical analyses were performed using IBM SPSS Statistics 22.0 (2013). After verification of the prerequisites, the data sets were compared by single-factor ANOVA. Normality of the data was checked by Kolmogorov-Smirnov test (when p>0.05, normal distribution) and homogeneity of variance was controlled by Levene test. If the variances were equal (p>0.05), Tukey’s b test was applied; if they were not equal (p<0.05), Dunnett’s T3 post hoc test was used. Correlation analysis of the parameters detected under the various light conditions and data representation was performed using Microsoft Office 365 (2016) Excel. Five free amino acids, Glu, Pro, Arg, Asp and Ser, were included into the correlation analysis. They were selected based on their central role in the metabolic pathways and their important biological functions. The hierarchical clustering was done by the Multi Experiment Viewer (v.4.5).

## Results

### Growth and photosynthesis

**Fig 1:**
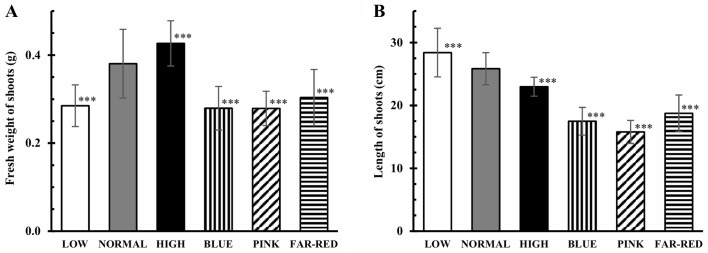
Effect of light conditions on fresh weight (A) and length (B) of shoots. After 5 days germination between wet filter papers, the plants were cultivated under six different light conditions. Light intensities and spectra are given in S1 Fig. Values indicated with asterisks are significantly different from those of detected in normal light at p≤0.01 (***) level. Three independent experiments (n = 12 in each experiment) were conducted.

Under increasing light intensity, the fresh weight of the shoots increased while their length decreased (Fig 1). Both parameters were significantly smaller in blue, pink and far-red lights compared to the normal white light. The fresh weight had high (0.7<r^2^<0.9) or very high (r^2^>0,9) positive correlation with GSH, Glu and Ser contents and the expression of most examined glutathione and amino acid metabolism-related genes (except for *APSR*, *NR*, *OrnATF*) and high negative correlation with the percentage of GSSG in the total thiol pool when the values of these parameters were analysed under various light conditions (S2 Table). The shoot length exhibited high or very high positive correlation with the expression of *GST*, *NR*, *SerMTF*, and high or very high negative correlation with pigment, GSSG and Asp contents and the transcript level of *APSR*.

**Fig 2:**
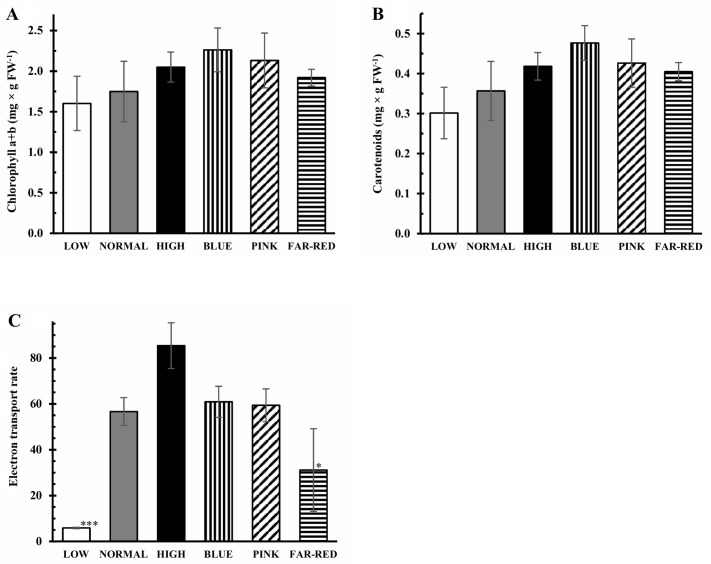
Effect of light conditions on the concentrations of chlorophyll (A) and carotenoids (B) and the photosynthetic electron transport rate (C). After 5 days germination between wet filter papers, the plants were cultivated under six different light conditions. Light intensities and spectra are given in S1 Fig. Values indicated with asterisks are significantly different from those of detected in normal light at p≤0.5 (*) and p≤0.01 (***) levels. Three independent experiments (n = 3 for the pigments and n = 10 for the electron transport rate in each experiment) were conducted.

The changes in the growth parameters may derive from the effect of light on the photosynthetic activity as indicated by the moderate correlation (r^2^ = 0.6) between fresh weight and ETR (S2 Table). The total chlorophyll content and the amount of carotenoids were 30% and 37% greater in blue light compared to normal white light, respectively (Fig 2A and 2B). The ETR increased significantly at higher light intensities and it was lower in far-red light than in normal white light (Fig 2C). The ETR showed high positive correlation with CySS, GSH and GSSG contents, the percentage of CySS in total cysteine pool and the expression of *OrnATF* (S2 Table).

### Thiol contents and their redox state

**Fig 3:**
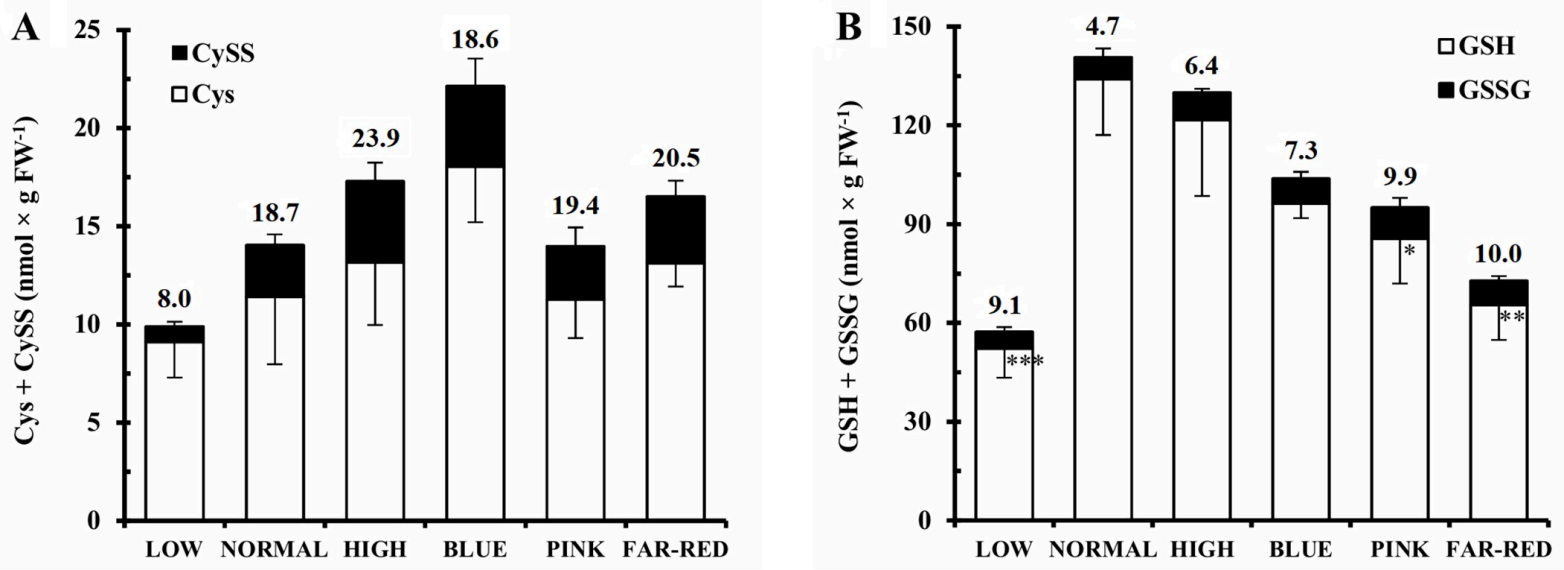
Effect of light conditions on cysteine and glutathione contents and redox state of glutathione. A: cysteine (Cys, white part of the columns) and cystine (CySS, black part of the columns), B: reduced (GSH, white part of the columns) and oxidised (GSSG, black part of the columns) glutathione. The numbers above the columns indicate percentage of CySS and GSSG in comparison to the total thiol pool (Cys+CySS and GSH+GSSG). After 5 days germination between wet filter papers, the plants were cultivated under six different light conditions. Light intensities and spectra are given in S1 Fig. Values indicated with asterisks are significantly different from those of detected in normal light at p≤0.5 (*), p≤0.1 (**) and p≤0.01 (***) levels. Three independent experiments (n = 3 in each experiment) were conducted.

The modification of photosynthesis affects the accumulation of reactive oxygen species and subsequently the level of antioxidants as shown by the above mentioned correlation between ETR and GSSG or GSH. The concentration of Cys, which is a precursor of GSH, thus being an important antioxidant, was 1.6-fold greater in blue light compared to normal white light (Fig 3A). Regarding the amount of CySS, the oxidised form was 75% less in low light than in normal light (Fig 3A). The total cysteine pool (Cys+CySS) contained about 20% CySS, except for its 8% value in low light. Both Cys and CySS contents showed high positive correlation with pigment and Asp concentrations and the expression of *APSR* gene and high negative correlation with the transcription of the *NR* gene (S2 Table). In addition, Cys exhibited high positive correlation with CySS content and the CySS concentration with ETR. The percentage of CySS showed high positive correlation with carotenoid and GSSG contents and ETR.

The GSH concentration was minimum 2.4-fold greater at the higher light intensities compared to low light (Fig 3B). Its level was significantly lower in pink and far-red lights compared to the normal white light. The concentration of glutathione disulphide (GSSG) was not affected by the light conditions. The glutathione-dependent redox state of the leaf tissues was characterised by the percentage of GSSG in the total glutathione pool (GSH+GSSG), which was at least 2-fold greater in low, pink and far-red light and also increased in high and blue light compared to the normal white light (Fig 3B). The GSH content was in high positive correlation with shoot fresh weight, ETR, Ser content and the expression of *CysASTL*, *GS2*, *APX*, *GluDH*, *P5CR* and *ArgSUL* genes (S2 Table). GSSG had high positive correlation with pigment and Asp contents and ETR and high negative correlation with shoot length. The percentage of GSSG showed moderate negative correlation with ETR and Glu contents and *AspTA* and *SerHMTF* transcript levels, high negative correlation with the expression of most other investigated genes except for the very high correlation with *GluDH* and *ArgSUL* genes and no correlation with *APSR* and *NR* genes.

### Pattern and concentration of free amino acids

Glutathione-dependent changes in the redox state of leaf tissues may affect amino acid metabolism because of the redox sensitivity of certain-related enzymes and this influence is indicated by the correlations described in the previous paragraph. In addition, GSH is a tripeptide and the use of Glu, Cys and Gly for its synthesis may influence the concentration of these amino acids.

**Fig 4:**
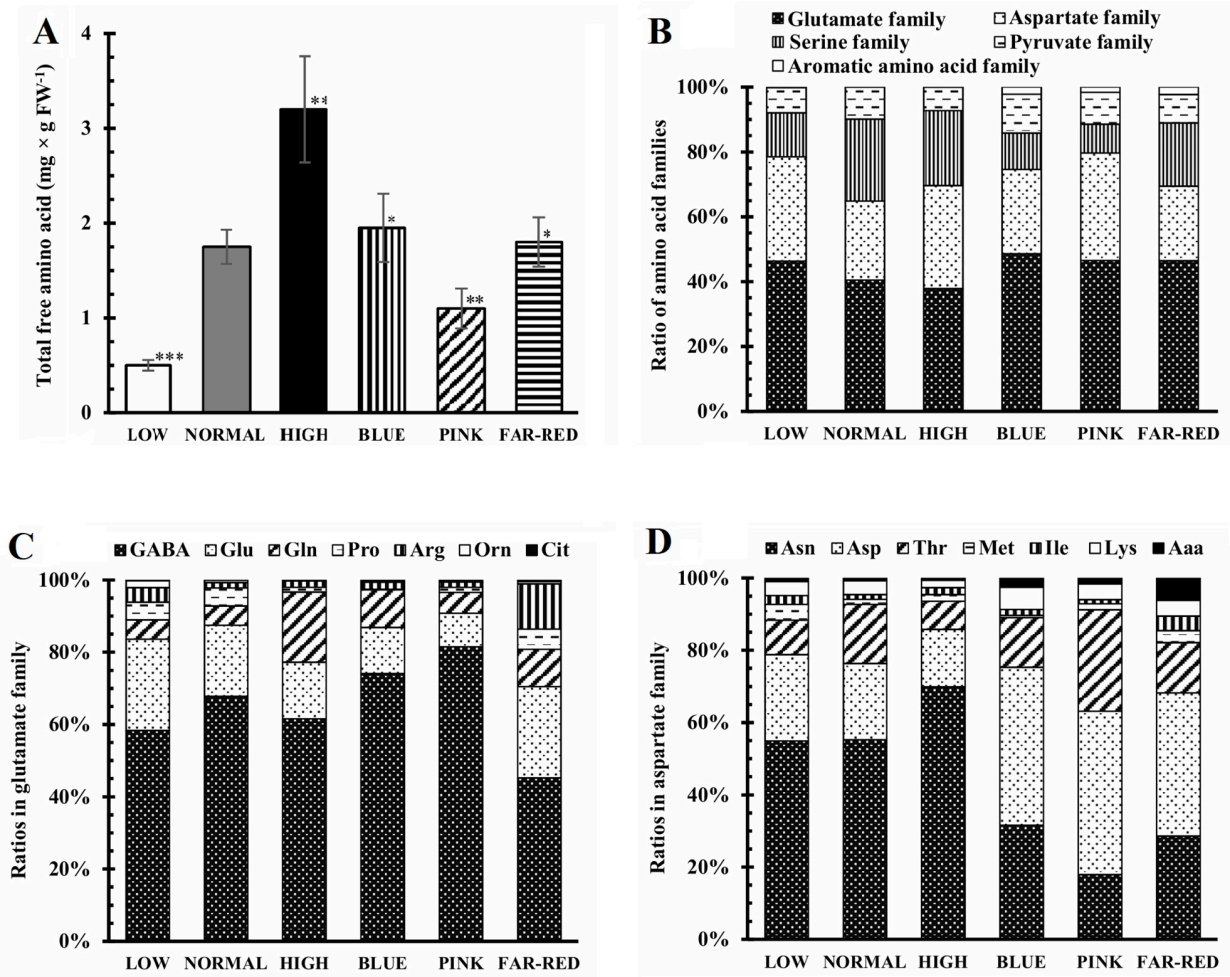
Effect of light conditions on total free amino acid content and the free amino acid patterns. A: total free amino acid content, B: ratios of the various families, C and D: ratios of free amino acids in the glutamate and aspartate families. After 5 days germination between wet filter papers, the plants were cultivated under six different light conditions. Light intensities and spectra are given in S1 Fig. Values indicated with asterisks are significantly different from those of detected in normal light at p≤0.5 (*), p≤0.1 (**) and p≤0.01 (***) levels. Three independent experiments (n = 3 in each experiment) were conducted.

The total free amino acid content increased significantly with growing light intensity and it was 36% lower in pink light than in normal white light (Fig 4A). The ratio of amino acid families was also affected by light conditions since the ratio of serine family was 50% lower in low, blue and pink lights compared to the other light conditions (Fig 4B). The ratio of glutamate and aspartate families altogether varied between 60 and80%: the smallest value was observed in normal light and the greatest in pink and low lights. In the glutamate family, both the light intensity and the spectral conditions greatly affected the ratio of the individual amino acids (Fig 4C). Manyfold changes were induced in the ratio of Gln, Pro, Arg and Orn. The high intensity and far-red lights had the greatest effects compared to the normal light. In the aspartate family, the ratio of Asn decreased greatly (from 55–75% to 20–30%) while the ratio of Asp increased considerably (from 15–20% to 40–45%) after the modification of the spectral composition compared to the normal white light (Fig 4D).

**Fig 5:**
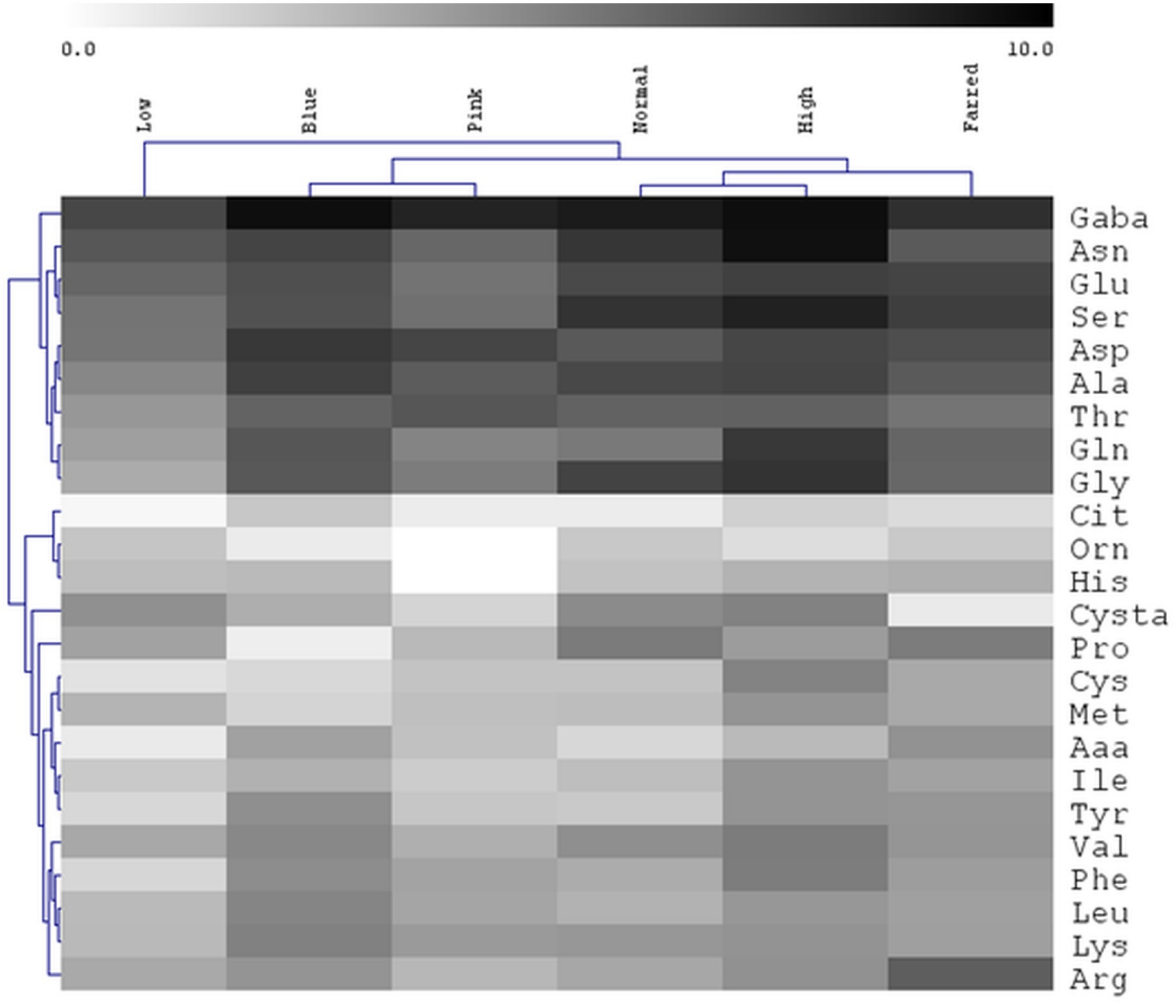
Hierarchical clustering of free amino acid concentrations detected under various light conditions. After 5 days germination between wet filter papers, the plants were cultivated under six different light conditions. Light intensities and spectra are given in S1 Fig. Three independent experiments (n = 3 in each experiment) were conducted.

The hierarchical clustering indicated that the concentration pattern of the individual amino acids greatly differed (separate cluster) in low light from all other light conditions (Fig 5; S3 Table). This pattern exhibited a large difference in blue and pink lights compared to normal, high and far-red lights. The greatest concentrations of many amino acids were detected in high light. Their amount grew with increasing light intensity except for Pro, Met and Orn. In pink light, the concentration of several amino acids was significantly smaller but the amount of Asp and Thr was greater compared to the normal intensity white light. Considering the clustering based on the concentrations of the individual amino acids, two main groups were formed. In one of them, the following amino acids occurred in large concentrations under nearly all light conditions: GABA, Asn, Glu, Ser, Asp. Ala, Thr, Gln, Gly. Among the other amino acids, the amounts of Cit and Orn were very low independently from the differences in light intensity and spectrum. Interestingly, very large concentration of Arg was detected in far-red light. Within one amino acid family, some amino acids were similarly, other ones were differently influenced by the changing light conditions. Thus, in glutamate family, Glu and Gln were similarly affected by the light condition while changes in Arg and Pro differed from them. The spectrum had a large effect on Pro and had no influence on GABA in the glutamate family.

The Glu content had high positive correlation with shoot fresh weight and Ser content (S2 Table). It exhibited a moderate positive correlation with all other parameters except for GSSG%, Asp content and *APSR*, *NR* and *SerHMTF* transcript levels. The concentration of Pro was in moderate negative correlation with the amount of pigments and in moderate positive one with the Glu content and the expression of *GS2*, *APX1*, *GST* and *SerHMTF* genes. The Arg level had moderate positive correlation with GSSG%, Glu and Pro levels and *APSR* expression while it showed moderate negative correlation with the transcription of *CysASTL*, *GluDH* and *ArgSUL* genes. The amount of Asp showed high positive correlation with Cys, CySS and GSSG contents and *APSR* expression and very high one with pigments. It had high negative correlation with shoot length and *NR* transcript level. Ser concentration was in positive correlation with most examined parameters, which ranged from moderate to very high level.

### Expression of genes related to glutathione and amino acid metabolism

**Fig 6:**
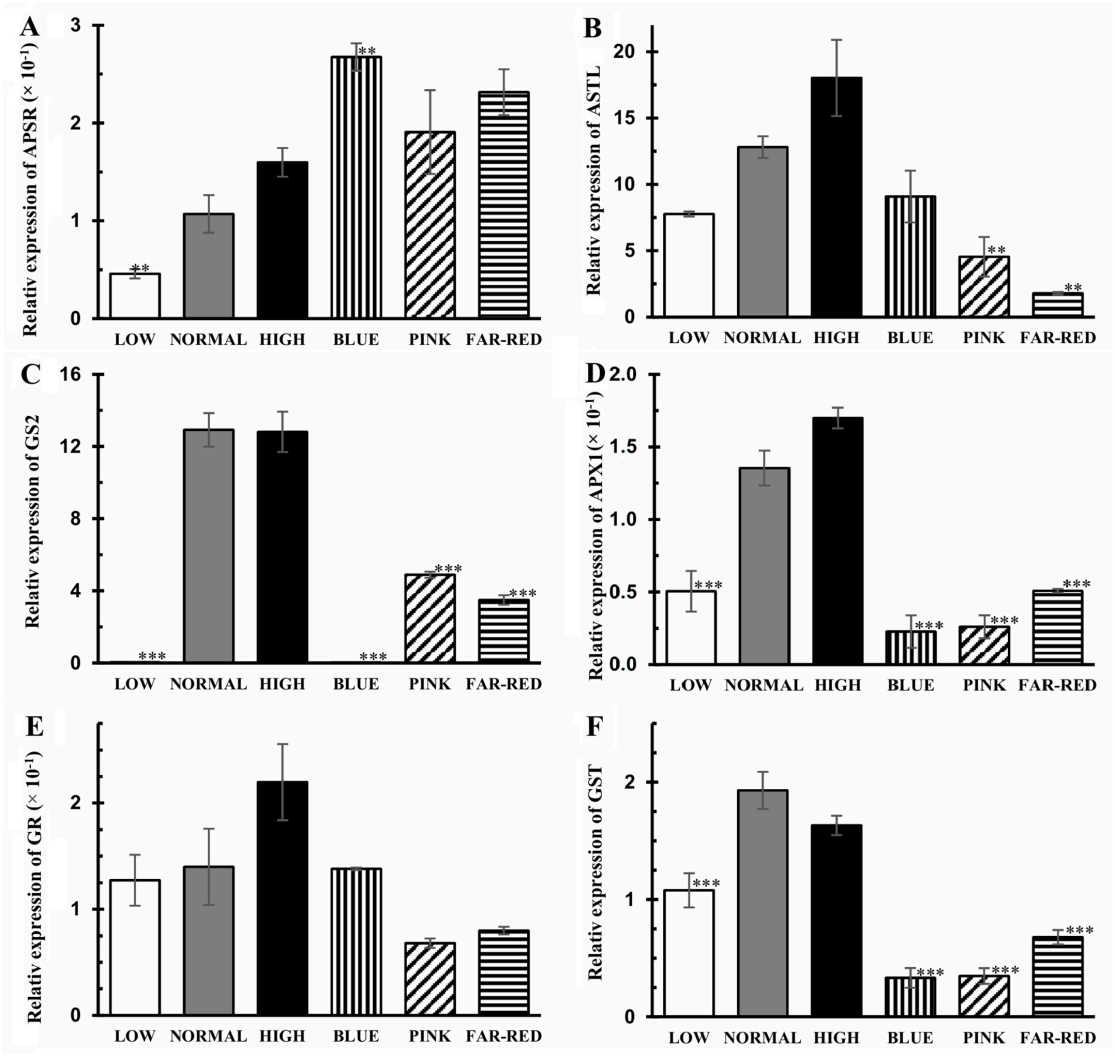
Relative expression of the genes related to the glutathione metabolism. A: adenosine phosphosulfate reductase (APSR), B: *O*-acetylserine (thiol) lyase (ASTL), C: glutathione synthase2 (GS2), D: ascorbate peroxidase1 (APX1), E: glutathione reductase (GR), F: glutathione *S*-transferase (GST). After 5 days germination between wet filter papers, the plants were cultivated under six different light conditions. Light intensities and spectra are given in S1 Fig. Values indicated with asterisks are significantly different from those of detected in normal light at p≤0.1 (**) and p≤0.01 (***) levels. Three independent experiments (n = 3 in each experiment) were conducted.

Alterations in the redox state of glutathione may modify the expression of genes encoding enzymes of glutathione metabolism and this assumption is supported by the previously mentioned negative correlation of the GSSG% with their transcription. The transcript level of the genes encoding APSR (key enzyme of sulfate reduction leading to the synthesis of Cys, the precursor of GSH), ASTL (last enzyme of Cys synthesis), GS2, APX1 (reduction of GSSG in the AsA-GSH cycle), GST (detoxification of organic peroxides and xenobiotics through their conjugation with GSH) was at least 40% greater in normal light compared to low light (Fig 6). The gene expression of GS2, APX1 and GST enzymes decreased at least by 60% in blue, pink and far-red lights compared to normal intensity white light. A large reduction (65% and 86%) in the transcript level of the *ASTL* gene was induced by pink and far-red lights.

**Fig 7:**
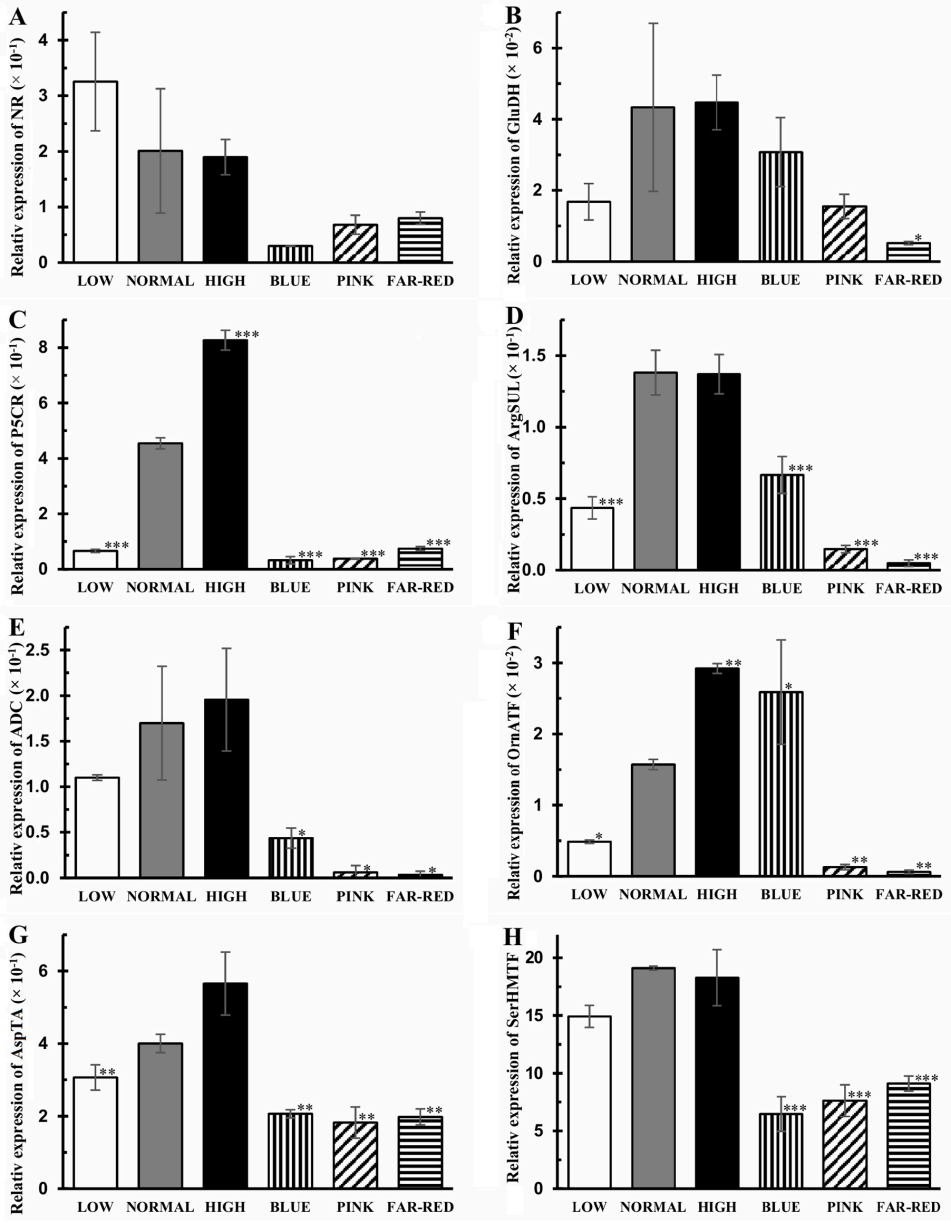
Relative expression of the genes related to the metabolism of amino acids. A: nitrate reductase (NR), B: glutamate dehydrogenase (GluDH), C: pyrroline-5-carboxylate reductase (P5CR), D: argininosuccinate lyase (ArgSUL), E: arginine decarboxylase (ADC), F: ornithine aminotransferase (OrnATF), G: aspartate transaminase (AspTA), I: serine hydroxymethyl-transferase (SerHMTF). After 5 days germination between wet filter papers, the plants were cultivated under six different light conditions. Light intensities and spectra are given in S1 Fig. Values indicated with asterisks are significantly different from those of detected in normal light at p≤0.5 (*), p≤0.1 (**) and p≤0.01 (***) levels. Three independent experiments (n = 3 in each experiment) were conducted.

Sulfate and nitrate reduction are interconnected through *O*-acetylserine; therefore, their co-ordinated, redox-dependent control under changing light conditions can be supposed. Inconsistence with this hypothesis, very high negative correlation (r^2^ = -0.96) was found between the expression of the genes (NR and APSR) encoding the key enzymes of the two pathways. While the expression of *NR* gene was not significantly affected by light intensity, the transcript level of several genes associated with the free amino acid metabolism increased (Fig 7). Thus, the expression of the genes encoding *P5CR* (Pro synthesis), *ArgSUL* (Arg synthesis), *OrnATF* and *AspTA* was significantly greater (6.3-, 3.3-, 3.6-, 1.3-fold greater) in normal light compared to low light. The transcript levels of *P5CR*, *ArgSUL*, *ADC*, *AspTA* and *SerHMTF* genes were significantly smaller (at least by 50%) in blue, pink and far-red lights than in normal white light (Fig 7). Interestingly, the expression of *OrnAT* gene was significantly greater in blue and significantly lower in pink and far-red lights compared to white light.

The expression of the most investigated glutathione and amino acid metabolism-related genes had high or very high positive correlation in their expression with each other (except for *APSR* and *NR*), which indicates their co-ordinated adjustment to the actual light intensity and spectrum (S2 Table). This co-ordination can be redox-based since the transcript level of most genes exhibited a positive correlation with the GSH content and negative one with the percentage of GSSG. The *APSR* and *NR* genes did not exhibit high correlations with the other genes except for the *HMTF* gene. *APSR* and *NR* had high or very high correlation with shoot length, pigment, Cys, CySS, GSSG and Asp contents, but the direction of the correlation was positive for *APSR* and negative for *NR*. The transcript level of the other genes showed no or only moderate correlations with these parameters.

## Discussion

### Influence of light conditions on growth and photosynthesis

Modifications in light condition affect the efficiency of photosynthesis and indirectly the availability of carbohydrates and energy for growth. Accordingly, the weight of the shoots of wheat seedlings grew with increasing light intensity (Fig 1A) as observed earlier in rape [34]. Similarly to *Oenothera* [13], low R/FR ratio decreased the shoot fresh weight in wheat (Fig 1A). However, its changes did not influence the shoot weight of *Chrysanthemum* [7]. While blue, pink and far-red light had similar inhibitory effect on the growth of wheat at 2-leaf stage (Fig 1B), such influence was exhibited only by blue light, in the flag leaves of older plants [6]. These observations indicate that the effect of spectral changes on the growth parameters probably depends on the developmental stage of the plants and it can be species-specific as well.

Although the photosynthetic pigments were not significantly affected by the increase in light intensity in wheat (Fig 2), the electron transport was greatly induced similarly to *Arabidopsis* [35]. The electron transport rate, as observed in wheat (Fig 2C), was also greater in blue light than in far-red one in bean leaves [36]. This phenomenon can be explained by the fact that the blue light-induced photocurrent is absorbed both by carotenoid and chlorophyll pigments and the red light-induced one is absorbed exclusively by chlorophyll. The observed differences in ETR under various light conditions may influence the growth as indicated by the positive correlation (r^2^ = 0.6) between these parameters (S2 Table). This effect is probably mediated by glutathione on the basis of the correlations between fresh weight, ETR, GSH content and GSSG percentage.

### Effect of light on glutathione metabolism and redox environment

The light intensity- and spectral composition-dependent changes in the electron transport rate may influence the production of ROS, their removal by the antioxidant system and consequently the redox state in the plant tissues [17]. In accordance with this hypothesis, light induced alterations in the redox state of glutathione were shown by the altered percentage of GSSG (Fig 3B). The relationship between the ETR and the redox state of glutathione was indicated by the negative correlation between them (r^2^ = -0.53, S3 Table). The increase in the percentage of GSSG was mainly due to the decrease in GSH content. These changes derived from the effect of light on its synthesis since both the level of its precursor, Cys (Fig 3A), and the expression of the genes related to Cys (Fig 6A and 6B) and GSH formation (Fig 6C) were affected by light conditions. These results indicate the transcriptional regulation of GSH synthesis which assumption is also supported by the positive correlation values including other genes of its metabolism (S2 Table). Cys is present in far lower concentration than Glu and Gly, the two other precursors of the tripeptide (Fig 5); therefore, its availability is a rate-limiting factor in GSH synthesis in wheat. A coordinated adjustment of the whole antioxidant system to the altered light conditions at transcriptional level is shown by the fact that the expression of the genes encoding *GS2*, *APX1*, and *GST* exhibited similar pattern under the applied light intensities and spectrums in wheat (Fig 6). The effect of blue and red light on the expression of *GST* gene was also shown in previous studies [37].

Similarly to wheat, increased levels of GSH were detected in *Arabidopsis* in high light [17]. The reducing effect of low R/FR ratio on GSH content observed in bean [20] was also corroborated in wheat (Fig 3B). As in young leaves, blue light also decreased the GSH content in the flag leaves of wheat [6]; however, pink and far-red lights had such effect only in the seedlings indicating a developmental stage-specific effect. These results show that GSH, as an antioxidant, has an important role in the adaptation of plants to changing light conditions.

### Control of amino acid metabolism by light

Synthesis of GSH and amino acids is interconnected at the level of sulphate (ensuring sulfur for Cys and subsequently for GSH) and nitrate reductions (providing N for amino acids) through O-acetylserine. The very high negative correlation between the key enzymes, APSR and NR at transcriptional level is not surprising if the optimal light conditions are modified (leading to increased GSSG%), since the GSH accumulation should be increased under adverse environmental condition when there is no need for a high level of amino acids due to the reduced protein formation and growth.

The effect of light on the free amino acid metabolism may be mediated by the GSH-dependent redox changes since a negative correlation was observed between the percentage of GSSG and transcript levels of the related genes (S3 Table). In addition, similar differences were observed in GSH and free amino acid levels between the plants grown under various light conditions (Figs 3 and 5) which results are in close agreement with earlier observations about the redox regulation of amino acid accumulation obtained in *Arabidopsis* [32]. Treatment of wild type and mutant plants deficient in AsA or GSH with GSH, AsA, dithiothreitol (DTT) and H_2_O_2_ clearly showed the redox control of the free amino acid concentrations since addition of these compounds could compensate the effect of mutations. In addition, treatment of leaf discs with DTT (a strong reductant) resulted in activation of the genes involved in amino acid metabolism and simultaneously in changes in the amino acid concentrations in Arabidopsis [38].

While enrichment of the spectrum in red and far-red components did not affect the total amino acid content in wheat seedlings (Fig 4A), the growth of barley seedlings in 100% red light resulted in a 1.4-fold increase of this parameter [5]. In addition, the amount of the individual amino acids was differentially affected in wheat while it exhibited a similar increase in barley indicating the distinct effect of monochromatic red light and its enrichment. The effect of light spectrum on amino acid levels was also shown indirectly in Arabidopsis using phytochrome mutants since loss of the light-absorbing pigments modified the concentration of amino acids [39].

Special attention should be dedicated to the glutamate family because of its high ratio (40%, Fig 4B) and involvement of its amino acids in the stress response. Similarly to wheat, red light greatly inhibited the transcription of the genes associated with the metabolism of amino acids including the members of the glutamate family in rice [3]. The level of GABA, also being a signalling molecule in plants [40], was greatly affected by spectrum in wheat, which may indicate its participation in the light quality-dependent control of growth, development and stress response. However, its high concentration in wheat does not support this function. Besides GABA, Gln, Pro, Arg through Orn and GSH through γ-glutamylcysteine are also synthesised from Glu; therefore, this amino acid has a central role in the glutamate family. In addition, its signalling role was proposed in plants, too [41]. Similarly to the present study, light quality-dependent control of Glu metabolism was also shown in maize [42]. Among the amino acids formed from Glu, Pro is very important not only in the stress response but it also ensures a link to the redox system and redox regulation of amino acid metabolism. During its formation, NADPH is needed for the reduction of Glu to pyrroline-5-carboxylate and the latter one to Pro and the produced NADP^+^ can be an electron acceptor in photosynthesis preventing the electron flow to oxygen and the synthesis of ROS [43]. Consequently, Pro can modulate the intracellular redox environment as shown in mammalian cells [44]. While in blue light low Pro level and in far-red light high Pro content were found in wheat seedlings (Fig 5), the opposite relationship was found for the different light conditions in tomato fruits [25] indicating an organ-, species- and developmental stage-specific control of Pro concentration by the spectrum. Another Glu-derived amino acid, Arg as storage compound of N and precursor of polyamines and NO, is involved in the control of growth and stress response [45,46]. Arg may participate in the mediation of the effect of light on these processes since far-red light had considerable effect on Arg levels (Fig 5) and the expression of the genes related to its metabolism in wheat (Fig 7).

Interestingly, both in aspartate and serine family, being the largest ones (20–40%) besides the glutamate family, the amounts of the individual amino acids were differently affected by the light conditions. Thus, within the aspartate family, Asn was converted to Asp in wheat when the spectrum of light was changed, which had only smaller effect on the concentration of Lys, Thr, Met and Ile being synthesised from Asp (Fig 4D). In serine family, Ser and Gly levels were far higher in blue light compared to the pink. Such difference was not observed for Cys (Fig 5). Among the three amino acids in this family, Ser has an important role in the control of plant development and metabolism [47], which function may be influenced by the light spectrum on the basis of the present results.

Correlations between the effects of light conditions on the level of certain amino acids (Glu, Pro, Arg, Asp, Ser) and the transcripts of the related genes indicated the transcriptional control of the amino acid metabolism in wheat (S2 Table). This regulation is redox-dependent as shown by the relationship between the redox state of GSH (percentage of GSSG in the glutathione pool) and expression levels. The effect of spectrum (blue and red light) on the transcription of genes related to amino acid (Phe, Tyr and Asp) metabolism was also shown in spruce, however, the possible simultaneous changes in amino acid levels were not monitored [48].

## Conclusions

Light intensity- and spectrum-dependent changes in photosynthesis affect the redox system in wheat. The induced modifications in the amount and redox state of glutathione may be involved in the mediation of the effect of light on transcriptional regulation of glutathione and amino acid metabolism. This relationship is indicated by the positive correlation of GSH concentration and the negative correlation of the GSSG percentage with ETR, the level of the studied metabolites or transcripts. It means that with the increase of GSH concentration, these parameters will also be greater, while with an increase of the GSSG, resulting in a more oxidising environment in the tissues, they will become smaller.

## Supporting information

S1 Table Primers used for qRT-PCR analysis. (XLSX)

S2 Table Correlation between the various examined parameters. (XLSX)

S3 Table Free amino acid content of wheat under different light conditions. *: significantly different from the value detected in normal light at P<0.05.
(DOCX)

S1 Fig Cultivation of the plants under different light conditions. Detailed data about light intensity and spectral conditions.
(TIF)
