# Supplementary material for: Light intensity and spectrum affect metabolism of glutathione and amino acids at transcriptional level
Source: PLoS One. 2019 Dec 31;14(12):e0227271. doi: 10.1371/journal.pone.0227271 (PMC6938384; doi:10.1371/journal.pone.0227271)
Supplement: S3 Table — *: significantly different from the value detected in normal light at P<0.05. (DOCX) [file pone.0227271.s003.docx]

| **FAA (µg/g)** | Low | | *Normal* | | High | | Blue | | Pink | | Far-red | |
| --- | --- | --- | --- | --- | --- | --- | --- | --- | --- | --- | --- | --- |
|  | Avg. | Std. | *Avg.* | *Std.* | Avg. | Std. | Avg. | Std. | Avg. | Std. | Avg. | Std. |
| Aaa | 1,79 | 0,24 | *3,00* | *0,42* | 6,63* | 0,53 | 13,11* | 1,90 | 5,50 | 0,69 | 19,48* | 1,01 |
| Ala | 26,45* | 1,18 | *147,76* | *5,59* | 165,20 | 1,30 | 181,93* | 9,61 | 84,13* | 2,79 | 89,01* | 13,04 |
| Arg | 10,44 | 0,55 | *10,98* | *1,83* | 20,25 | 2,98 | 18,41 | 3,15 | 7,22 | 1,45 | 79,64* | 14,34 |
| Asn | 98,33* | 9,10 | *242,15* | *4,80* | 677,63* | 19,72 | 161,97* | 19,98 | 63,23* | 3,37 | 90,21* | 19,74 |
| Asp | 42,89* | 2,27 | *92,40* | *2,77* | 154,25* | 3,98 | 224,34* | 11,24 | 159,25* | 17,73 | 125,25* | 2,46 |
| Cit | 0,54 | 0,12 | *0,73* | *0,01* | 0,85 | 0,04 | 4,62* | 0,56 | 1,68* | 0,18 | 2,69* | 0,26 |
| Cys | 0,44 | 0,03 | *0,77* | *0,03* | 0,91 | 0,02 | 2,95* | 0,23 | 5,11* | 0,22 | 10,50* | 0,81 |
| Cysta | 20,90 | 1,30 | *23,97* | *2,10* | 30,12* | 2,61 | 9,09* | 0,69 | 3,30* | 0,46 | 1,80* | 0,05 |
| GABA | 150,91* | 6,97 | *494,66* | *2,16* | 710,70* | 7,20 | 709,25* | 12,83 | 402,61* | 30,77 | 289,21* | 8,37 |
| Gln | 13,89* | 1,84 | *39,07* | *3,42* | 223,80* | 9,84 | 99,58* | 10,97 | 28,88 | 4,40 | 66,12* | 10,51 |
| Glu | 65,27* | 5,70 | *143,16* | *5,36* | 180,83* | 4,26 | 122,45 | 0,78 | 45,68* | 11,61 | 161,61 | 17,17 |
| Gly | 10,04* | 1,64 | *167,32* | *7,96* | 257,29* | 14,51 | 94,20* | 4,52 | 35,92* | 1,56 | 63,81* | 11,05 |
| His | 5,92 | 0,27 | *5,29* | *0,15* | 8,08* | 0,14 | 6,56 | 1,18 | 0,88* | 0,12 | 8,94* | 1,56 |
| Ile | 4,41 | 0,20 | *5,92* | *0,43* | 18,33* | 0,46 | 8,41* | 0,74 | 4,06 | 0,63 | 12,76* | 1,67 |
| Leu | 6,54 | 1,17 | *8,21* | *0,24* | 17,27* | 0,26 | 27,86* | 1,38 | 11,61* | 0,87 | 13,57* | 2,00 |
| Lys | 6,84* | 0,21 | *16,98* | *2,06* | 19,12 | 1,96 | 31,37* | 2,02 | 15,65 | 0,95 | 13,72 | 2,00 |
| Met | 7,86 | 0,36 | *6,07* | *0,22* | 18,43* | 2,38 | 3,28 | 0,28 | 5,70 | 1,54 | 10,47* | 1,55 |
| Orn | 4,90 | 0,69 | *4,52* | *0,42* | 2,52* | 0,29 | 1,77* | 0,07 | 1,00* | 0,08 | 4,40 | 0,15 |
| Phe | 0,33 | 0,02 | *0,48* | *0,01* | 1,09 | 0,02 | 22,63* | 2,60 | 12,26* | 1,47 | 14,39* | 2,80 |
| Pro | 12,69* | 1,37 | *36,21* | *3,10* | 14,85* | 1,09 | 1,62* | 0,34 | 6,87* | 0,36 | 36,08 | 1,89 |
| Ser | 44,24* | 5,25 | *261,74* | *7,18* | 418,91* | 7,82 | 115,95* | 7,18 | 49,24* | 8,73 | 192,34* | 12,82 |
| Thr | 17,02* | 1,95 | *71,50* | *2,52* | 75,22 | 5,11 | 70,39 | 2,58 | 99,04* | 11,97 | 44,05* | 12,84 |
| Tyr | 0,33 | 0,02 | *0,49* | *0,02* | 1,17 | 0,03 | 21,49* | 1,02 | 4,74 | 0,59 | 17,65* | 4,69 |
| Val | 10,74* | 1,88 | *21,23* | *1,66* | 35,62* | 1,36 | 25,46 | 3,12 | 9,00* | 1,53 | 17,93 | 3,13 |

**S3 Table. Free amino acid content of wheat (µg/g FW).**

*Values indicated with asterisks are significantly different from those of detected in normal light (the data sets were compared by single-factor ANOVA followed by Tukey's post hoc test, p<0.05 level).
