# Supplementary figures and images for: Light intensity and spectrum affect metabolism of glutathione and amino acids at transcriptional level
Source: PLoS One. 2019 Dec 31;14(12):e0227271. doi: 10.1371/journal.pone.0227271 (PMC6938384; doi:10.1371/journal.pone.0227271)

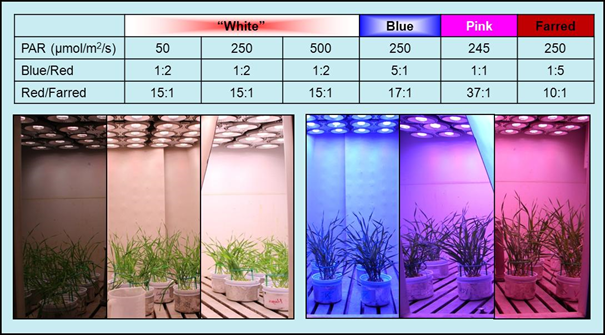

Supplement: S1 Fig — Detailed data about light intensity and spectral conditions. (TIF) [file pone.0227271.s004.tif]
